# Application of 3D and 2D quantitative shear wave elastography (SWE) to differentiate between benign and malignant breast masses

**DOI:** 10.1038/srep41216

**Published:** 2017-01-20

**Authors:** Jie Tian, Qianqi Liu, Xi Wang, Ping Xing, Zhuowen Yang, Changjun Wu

**Affiliations:** 1 Ultrasound Department, the First Affiliated Hospital of Harbin Medical University, Harbin, China; 2 Endocrinology Department, the First Affiliated Hospital of Harbin Medical University, Harbin, China

## Abstract

As breast cancer tissues are stiffer than normal tissues, shear wave elastography (SWE) can locally quantify tissue stiffness and provide histological information. Moreover, tissue stiffness can be observed on three-dimensional (3D) colour-coded elasticity maps. Our objective was to evaluate the diagnostic performances of quantitative features in differentiating breast masses by two-dimensional (2D) and 3D SWE. Two hundred ten consecutive women with 210 breast masses were examined with B-mode ultrasound (US) and SWE. Quantitative features of 3D and 2D SWE were assessed, including elastic modulus standard deviation (E_SD_^E^) measured on SWE mode images and E_SD_^U^ measured on B-mode images, as well as maximum elasticity (E_max_). Adding quantitative features to B-mode US improved the diagnostic performance (*p* < 0.05) and reduced false-positive biopsies (*p* < 0.0001). The area under the receiver operating characteristic curve (AUC) of 3D SWE was similar to that of 2D SWE for E_SD_^E^ (*p* = 0.026) and E_SD_^U^ (*p* = 0.159) but inferior to that of 2D SWE for E_max_ (*p* = 0.002). Compared with E_SD_^U^, E_SD_^E^ showed a higher AUC on 2D (*p* = 0.0038) and 3D SWE (*p* = 0.0057). Our study indicates that quantitative features of 3D and 2D SWE can significantly improve the diagnostic performance of B-mode US, especially 3D SWE E_SD_^E^, which shows considerable clinical value.

Breast cancer is one of the chief causes of death among women worldwide^1^. Ultrasound (US), as an irreplaceable tool in breast imaging and an adjunct to mammography^2^, is also used as a first-line examination to both detect and characterize breast masses. The Breast Imaging Reporting and Data System (BI-RADS) with US^3^ provides a standardized terminology for describing and classifying breast masses, and satisfactory diagnostic performance has been recently reported^4^. However, conventional US has an important limitation, i.e., low specificity, as reported in previous research^5^,^6^. Recent studies have demonstrated that US elastography developed in the past decade can improve the diagnostic performance of breast US, including its specificity^7^,^8^.

Malignant breast masses are generally stiff, and benign masses are generally soft^9^. US elastography is a new diagnostic imaging method for detecting the degree of tissue stiffness. Recent studies have demonstrated that strain elastography, an important adjunct to US^10^,^11^, shows notable diagnostic performance in differentiating benign and malignant breast lesions, with a sensitivity of 78–98.6% and a specificity of 51.7–98.5%^12^,^13^,^14^,^15^,^16^. However, as the elasticity map obtained is highly dependent on the extent of tissue compression and on the tissue’s compressibility limits under stress, the quality and reliability of strain elastography are affected considerably by interobserver variability during data acquisition and interpretation^17^. In addition, the acquired information is a qualitative rather than a quantitative measurement of local strain, rendering the efficacy of the modality a matter of debate.

Shear wave elastography (SWE), a quantitative imaging technique, has been developed to address the above problems. Reportedly, SWE decreases the operator dependence and shows high reproducibility^18^,^19^,^20^. This technique allows the measurement of the propagation speed of shear waves within tissues in metres per second (m/s) to locally quantify tissue stiffness (Young’s modulus) in kilopascals (kPa). Several studies have reported the diagnostic application of SWE to differentiating breast mass types^7^,^21^,^22^,^23^. These studies have shown that the addition of SWE parameters can improve the diagnostic accuracy of breast US^7^,^8^,^24^,^25^. Moreover, according to Evans^26^, SWE elasticity values in breast cancer tissues show a significant association with prognostic factors, including tumour invasion, type and histological grade.

However, most studies of SWE have focused on two-dimensional (2D) images; thus, the main SWE images for breast masses have been obtained on a single imaging plane. As a result, the information on the elasticity of breast lesions is far from being exhaustively evaluated. To address this issue, three-dimensional (3D) SWE has been introduced for breast US diagnosis. This technology can reconstruct volumetric images from data obtained from a single sweep of the US beam across the entire area of interest. In 3D SWE, comprehensive elastographic information about all aspects of masses can be obtained. In addition, the heterogeneity of the whole mass can be viewed in three dimensions. The stiffest position within a mass can be effectively observed, and various stiffness values can be quantitatively measured. 3D SWE has been shown to provide acceptable interobserver agreement^27^. Lee *et al*.^28^ reported that the quantitative factors of both 2D and 3D SWE, such as the maximum stiffness (E_max_), stiffness ratio (E_rat_) and mean stiffness (E_mean_), have significantly improved the diagnostic performance of breast US. However, the evaluation of these quantitative factors is susceptible to disturbances during 3D SWE image acquisition that can cause artefacts^28^. To the best of our knowledge, few studies have reported on how to assess the standard deviation (SD) values in 3D SWE. Additionally, the measurement methods used for assessing SD values have been inconsistent in different studies^29^,^30^.

The objective of our study was to analyse the diagnostic value of SD measured by different methods and to evaluate the diagnostic performance of elasticity features (E_max_ and SD) quantified by 2D and 3D SWE to distinguish benign from malignant breast masses.

## Materials and Methods

### Patients and masses

This retrospective study was conducted with approval from the Institutional Review Board of the First Affiliated Hospital of Harbin Medical University, and informed patient consent was waived. The methods in our study were applied in accordance with approved guidelines. From March 2014 to May 2015, 248 consecutive women were examined with B-mode US and both 2D and 3D SWE before undergoing US-guided core needle biopsy (CNB) or surgical breast mass excision. The exclusion criteria were as follows: women with breast implants; women who previously underwent breast surgery and had a family history of breast cancer; women who were pregnant or breast feeding; women whose masses were categorized as BI-RADS 0 or 6^3^; and women whose masses were larger than the maximum width of the SWE colour overlay, which was 3 cm. One lesion per patient was included. The inclusion criteria for multiple breast masses were as follows: if multiple masses were present, that with the most suspicious morphological features was evaluated by B-mode US; if all the masses showed malignant morphological features, a randomly selected mass was evaluated; if all the masses showed benign morphological features, a randomly selected mass was evaluated. A total of 210 solid breast masses in 210 women aged from 18 to 80 years (mean, 43.12 ± 13.34 years) were included in this study. One hundred fifty-one (71.9%) of these women were symptomatic, including 112 patients with a palpable mass, 13 with nipple discharge and 41 with breast pain. One hundred sixty-eight breast masses were visible on mammograms.

The histopathological evaluation of the breast lesions was performed by a pathologist with 20 years of experience in breast pathology. US-guided CNB was initially performed for breast masses assigned BI-RADS categories of 5 and 4, and masses assigned BI-RADS categories of 3 and 2 were evaluated at the request of the patient or referring physician. After CNB, some benign breast masses were excised depending on the preferences of the physician and patient. For masses with benign biopsy histological findings, six to eight months of US follow-up was recommended to confirm the mass stability. The histopathology of the 210 breast masses was confirmed via US-guided CNB (n = 112) and surgery (n = 98).

### US examination

B-mode US and SWE were performed using an Aixplorer US system (SuperSonic Imagine, Aix-en-Provence, France) by one of three experienced radiologists. 2D US and SWE images were acquired using a 4- to 15-MHz linear-array transducer. 3D SWE images were acquired using a 5- to 16-MHz dedicated mechanical volumetric transducer. The greyscale US features of the breast masses were assessed on 2D US images acquired on 2 orthogonal planes, according to the BI-RADS US. The longest diameter of each mass was measured on the B-mode images.

After B-mode US, 2D and 3D SWE acquisitions were performed for the breast masses that were scheduled to be biopsied or excised. During image acquisition, the probe was maintained perpendicular to the skin and kept still for a few seconds to stabilize the SWE images^31^. To reduce artefactual stiffness, after the probe was applied with the slightest pressure and a generous layer of US gel, the patients were instructed to hold their breath. A square region of interest (ROI) set for SWE acquisition was adjusted to include the whole mass and surrounding normal tissue observed by B-mode, excluding the skin and chest wall. In the ROI, stiffness was displayed as a semi-transparent colour-overlaid map using the default stiffness range from dark blue to red (0–180 kPa). For 2D SWE, masses were observed by different cross-sectional views. SWE images were chosen and saved in top-bottom display format for retrospective analysis. The total examination was accomplished in less than 5 minutes, including the 2D B-mode US examination. For 3D SWE, the SuperSonic 3D breast probe acquired 2D images and reconstructed a 3D image from the 2D data. Volumetric SWE data were reconstructed and displayed in multi-slice and multi-planar views by featuring the transverse, sagittal, and coronal planes. With a slow-tilt movement of the sectorial mechanical transducer and a sweep angle of 10–30°, volumetric imaging was automatically performed, and the data were saved as image files for later review. The 3D SWE examination was accomplished in less than 5 minutes.

### Image evaluation

**Figure 1:**
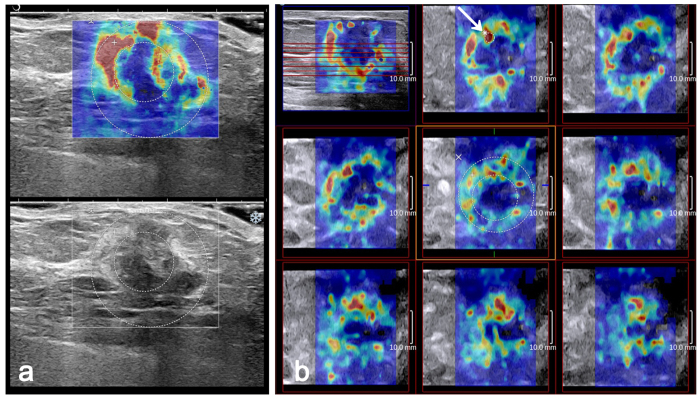
Invasive ductal carcinoma grade 2 in a 61-year-old woman. The 2D B-mode image shows an irregular hypoechoic mass considered to be BI-RADS category 4b (bottom). On 2D SWE (**a**) and 3D SWE (**b**), an ROI for measuring the E_SD_^E^ value was placed to include the whole breast mass and immediately adjacent stiff tissue. The other Q-Box for measuring the E_SD_^U^ was placed within the mass to encompass the maximum portion of the mass and exclude tissue outside the mass. (**a**) E_max_ value recorded using the larger Q-Box. (**b**) E_max_ value recorded using a 2-mm^2^ Q-Box (arrow).

All the images were reviewed by two radiologists together who were blinded to the clinical, mammographic and pathological findings. Disagreements were resolved by consensus. The E_max_ and SD of each mass were measured for the quantitative analysis. Three separate SWE acquisitions were performed for each mass, and the averages of the elasticity values were used in this analysis. In our study, SD was measured using two methods in both 2D and 3D SWE. The size of the ROI for the two measurement methods was adjusted according to the breast mass size. In one method, the built-in ROI (Q-Box^TM^; SuperSonic Imagine) adjusted to the mass contours on SWE-mode images was set to include the mass and immediate adjacent stiff tissue or halo. This calculated SD was recorded as E_SD_^E^. In the other method, a Q-Box adjusted to the mass contours on B-mode images was placed within the mass to encompass the maximum mass area. This calculated SD was recorded as E_SD_^U^ (Fig. 1). For 2D SWE, E_max_ was measured using the first method. For 3D SWE, quantitative variables were measured on all the three orthogonal planes as follows: on the multi-slice view, a 2-mm^2^ Q-Box was placed on the stiffest area of the mass or its adjacent periphery, and the E_max_ value was recorded (Fig. 1). Two SD measurements were performed on the same SWE image, which included the largest equatorial plane of the mass (Fig. 1).

There were five data sets, consisting of B-mode US, 2D SWE, 3D SWE, and combined sets of B-mode US and SWE data. According to the American College of Radiology (ACR)-BI-RADS, the second version for US, the final assessment category of each mass was recorded by the reviewers to indicate the probability of malignancy among categories 2, 3, 4, 4a, 4b, 4c, and 5^32^. According to the ACR guidelines, masses categorized as BI-RADS 4a or higher were recommended to undergo a biopsy, and masses categorized as BI-RADS 3 were recommended for follow-up only. The hypothetical effect of the combination of SWE with conventional US was evaluated to inform the management decision over whether to perform a biopsy. Another assessment was performed with combined sets of greyscale and SWE data, taking the B-mode US BI-RADS information as the starting point. Reviewers were asked to downgrade or upgrade masses and change their BI-RADS classification using the following rules: if SWE variables did not suggest malignancy (i.e., were below the cut off values), BI-RADS category 4a masses would be moved to BI-RADS category 3; if SWE variables did suggest malignancy (i.e., were equal to or larger than the cut off values), BI-RADS category 3 masses would be moved to BI-RADS category 4a. However, BI-RADS category 3 masses would not be downgraded, and BI-RADS category 4b or higher masses would not be upgraded.

### Statistical analysis

As continuous variables did not follow a normal distribution, the results are reported as medians and interquartile ranges (IQRs). Categorical variables were analysed in terms of frequencies and percentages. The quantitative 2D SWE and 3D SWE elasticity values (E_SD_^E^, E_SD_^U^ and E_max_) of benign and malignant masses were compared using the rank-sum test. Paired data of continuous variables in the same masses were compared using the paired rank-sum test. Receiver operating characteristic (ROC) analyses were performed to assess the diagnostic performance of the variables, determine the optimal cut off values using the Youden index (maximum of sensitivity + specificity − 1), and calculate the corresponding sensitivity and specificity. A logistic regression model was used for the ROC analysis of combined data sets (B-mode and SWE variables) and to compare the diagnostic performances of B-mode vs B-mode combined with 2D SWE and B-mode vs B-mode combined with 3D SWE using the area under the ROC curve (AUC) as the indicator of performance. The McNemar test was used to perform paired comparisons of proportions (sensitivity and specificity).

Statistical analyses were performed using dedicated commercial software (SAS, version 9.1.3; SAS, Cary, NC, USA). For multiple pairwise comparisons, *p* values less than the Bonferroni-corrected significance value of 0.05/9 = 0.0083 were used to indicate statistically significant differences. In other instances, *p* values less than 0.05 were considered to indicate statistical significance.

## Results

### Breast masses

There were 73 (34.76%) malignant masses and 137 (65.24%) benign masses detected in our study. Among the cancerous masses, 59 (28.1%) were infiltrating ductal carcinomas, 9 (4.28%) were ductal carcinomas *in situ* (DCIS), 2 (0.95%) were mucinous carcinomas, 1 (0.48%) was an invasive lobular carcinoma, and 2 (0.95%) were intraductal papillary carcinomas. Among the benign masses, 72 (34.28%) were fibroadenomas, 25 (10%) were fibroadenomatous hyperplasias, 8 (3.81%) were cystic hyperplasias, 11 (5.24%) were papillomas, 6 (2.86%) were adenosis, 7 (3.33%) were mammary duct ectasias, 6 (2.86%) were chronic inflammations, and 2 (0.95%) were fat necrosis. The maximal diameter of the masses by US was 17.04 ± 6.07 mm (range: 4.6–30 mm). The mean size of benign masses was 16.18 ± 5.92 mm (range: 4.6–28.9 mm), and the mean size of malignant masses was 18.95 ± 5.89 mm (range: 7–30 mm).

### Quantitative SWE features

E_SD_^E^, E_SD_^U^, and E_max_ were significantly higher in malignant masses than in benign masses on both 2D and 3D SWE (*p* < 0.0001 for all) (Table 1). E_SD_^E^ was significantly higher than E_SD_^U^ in both 2D and 3D SWE (*p* < 0.0001). A comparison of 2D with 3D SWE data revealed that E_max_ was significantly higher in 3D SWE than in 2D SWE for the same breast masses (*p* < 0.0001). Both E_SD_^E^ and E_SD_^U^ were significantly higher in 3D SWE than in 2D SWE for benign masses (*p* = 0.0294 and *p* = 0.0463, respectively), while no significant differences were found in malignant masses (*p* = 0.735 and *p* = 0.844, respectively).

### Diagnostic performance of SWE features

Table 2 summarizes the diagnostic performance of the 2D and 3D SWE features and the results of a comparison between the 2D and 3D SWE features. A comparison of E_SD_^E^ with E_SD_^U^ showed that the AUC of E_SD_^E^ was significantly higher than that of E_SD_^U^ in both 2D SWE (*p* = 0.0038) and 3D SWE (*p* = 0.0057). A comparison of E_SD_^E^ with E_max_ revealed that there were no significant differences in the AUC values either in 2D SWE (*p* = 0.0438) or in 3D SWE (*p* = 0.6459). A comparison of E_max_ with E_SD_^U^ showed that the AUC of E_max_ was significantly higher than that of E_SD_^U^ in 2D SWE (*p* = 0.0016), while there was no significant difference in 3D SWE (*p* = 0.0676). In a comparison of 2D and 3D SWE data, no significant differences were found in the AUC for either E_SD_^E^ (0.9553 vs 0.9306, *p* = 0.026) or E_SD_^U^ (0.9142 vs 0.8891, *p* = 0.159). E_max_ showed a higher AUC in 2D SWE than in 3D SWE (0.9636 vs 0.9252, *p* = 0.002). The specificity of E_max_ in 2D SWE was significantly higher than that in 3D SWE (*p* = 0.000), whereas there were no significant differences for E_SD_^E^ (*p* = 0.303) or E_SD_^U^ (*p* = 0.060) (Table 2).

### Diagnostic performance of B-mode US and B-mode US combined with SWE

The AUC of the BI-RADS assessments based on B-mode US alone was 0.9064, and the optimal cut off point was between categories 4a and 4b (Table 3). The corresponding sensitivity and specificity were 91.78% (61/73) and 80.92% (110/137), respectively. Adding SWE features to B-mode US significantly improved both the AUC (*p* < 0.05 for all) and specificity (*p* < 0.05 for all).

### Hypothetical effect of SWE on BI-RADS assessments

**Figure 2:**
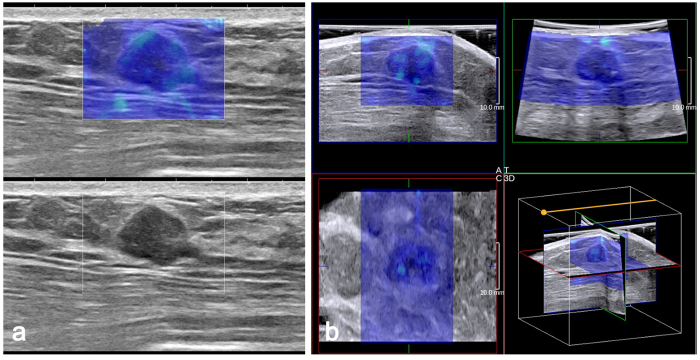
Fibroadenoma in a 45-year-old woman. (**a**) The 2D B-mode image shows a hypoechoic mass with parallel orientation considered to be BI-RADS category 4a (bottom). Both 2D SWE (**a**) and 3D SWE (**b**) show a homogeneously soft (blue colour) mass and surrounding tissue (2D E_max_ 47.6 kPa, E_SD_^E^ 8.0 kPa, E_SD_^U^ 7.7 kPa; 3D E_max_ 54.6 kPa, E_SD_^E^ 10.8 kPa, E_SD_^U^ 9.1 kPa). When combining SWE and B-mode US, BI-RADS category 3 was assigned to the mass.

Among the benign masses, 61.31% (84/137) were recommended to undergo an interventional procedure based on B-mode features only (Table 4). When the E_SD_^U^, E_SD_^E^ and E_max_ of 2D SWE were included, this percentage significantly decreased to 25.55% (35/137), 21.89% (30/137) and 21.16% (29/137), respectively (*p* < 0.0001 for all) (Fig. 2). When the E_SD_^U^, E_SD_^E^ and E_max_ of 3D SWE were included, this percentage significantly decreased to 29.92% (41/137), 23.35% (32/137), and 27.73% (38/137), respectively (*p* < 0.0001 for all). Among the malignant masses, 98.63% (72/73) were recommended to undergo an interventional procedure based on the B-mode features only. When the SWE features from both 2D and 3D SWE were included, this percentage decreased to 95.85% (70/73) or 97.26% (71/73), which were not significant differences.

## Discussion

In this study, quantitative elasticity features were compared between 2D and 3D acquisition techniques. The quantitative values were significantly higher in the malignant masses than in the benign masses on 3D and 2D SWE (*p* < 0.0001 for all), which was consistent with the results reported by other studies^8^,^27^. Berge *et al*.^7^ reported that the addition of SWE features to BI-RADS assessments could increase the AUC. Lee *et al*.^28^ showed that both 2D and 3D SWE could increase the AUC of diagnostic US imaging. Similarly, in our study, the quantitative elasticity values showed notable diagnostic performance in differentiating benign from malignant breast masses, and the addition of 3D and 2D SWE parameters to B-mode features significantly improved the AUC and specificity of B-mode US alone.

Other studies demonstrated that E_max_ was the quantitative SWE feature of the optimal performance for breast cancer diagnosis^7^,^33^. In our study, E_max_ showed remarkable performance, with an AUC of 0.943 in 2D SWE. However, the AUC and specificity of E_max_ in 3D SWE was notably lower than that in 2D SWE (*p* = 0.002 and *p* = 0.000). In our study, E_max_ in 3D SWE was significantly higher than that in 2D SWE for the same breast masses, which was consistent with the findings of a previous study^28^. Shear wave velocity is susceptible to external compression^34^. A heavier convex 3D probe may cause higher levels of pressure than a 2D probe^27^,^28^. The artefactual stiffness caused by external compression often appears close to the skin side of the elastic image. Using adequate US gel on the surface of the 3D probe and floating the probe gently on the surface of the skin may effectively reduce the occurrence of stress artefacts.

**Figure 3:**
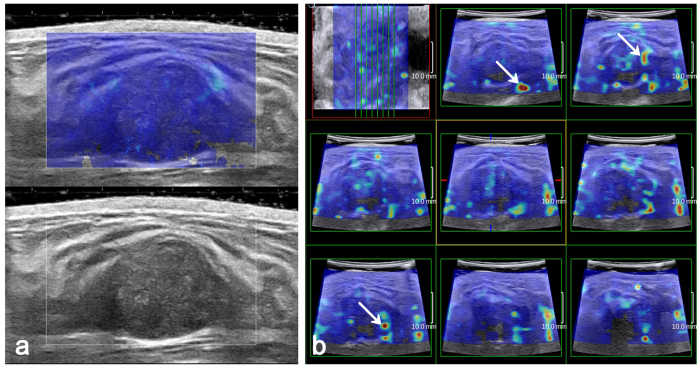
Fibroadenoma in a 29-year-old woman. (**a**) The 2D SWE shows a homogeneously soft (blue colour) mass and surrounding tissue at the margin of the breast mass (E_max_ 49.7 kPa). (**b**) Multi-slice 3D SWE images show a heterogeneously stiff mass with scattered red colour (arrow) (E_max_ 155 kPa).

In addition, 3D SWE images were obtained dynamically and automatically and were thus more susceptible to interference from artefacts, which are hereby named “dynamic artefacts”. These artefacts often appear as scattered spots on the SWE image. The artefactual stiffness may involve the centre and periphery of the breast masses, which may be another cause of the higher E_max_ in 3D SWE. In our study, there were 18 benign masses showing false-positive E_max_ in 3D SWE caused by the artefactual stiffness but negative E_max_ in 2D SWE (Fig. 3). The dynamic artefact may be caused by the potential technical error of 3D SWE. Therefore, these artefacts may be the factors that affect the stability of the E_max_ measurements in 3D SWE and may explain the lower E_max_ AUC and specificity in 3D SWE.

In this study, E_SD_^E^ showed a diagnostic accuracy comparable to that of E_max_, while E_SD_^U^ showed a lower AUC than that of E_max_ in 2D SWE (*p* = 0.0016). The heterogeneous colour feature was considered useful for differentiating benign from malignant breast lesions^25^. E_SD_ can serve as a measure of lesion heterogeneity^35^. In our data, SD showed good diagnostic performance in 2D SWE, which was consistent with the results of a previous study^25^. In addition, SD did not show any significant differences in diagnostic performance between 2D and 3D SWE. Consequently, the measurement of SD may be relatively stable and less susceptible to the dynamic artefacts of 3D SWE. Moreover, all the elastographic information of the 3D SWE volume could be visualized and measured. SD measured by 3D SWE may better reflect the heterogeneity of the whole tumour mass. In our data, one follow-up decision (category 3) was changed to a biopsy decision (category 4a) after the inclusion of E_SD_^E^ in both 2D and 3D SWE and E_max_ in 3D SWE. For the lower diagnostic performance of E_max_ in 3D SWE, this upgrade caused by E_max_ in 3D SWE may be distorted by dynamic artefact interference. Therefore, SD measurements may show better stability than E_max_ measurements in 3D SWE and can be effectively used for differentiating benign from malignant breast masses in 3D SWE.

In our study, E_SD_^E^ had a higher diagnostic value than E_SD_^U^. Compared with E_SD_^U^, E_SD_^E^ provided higher AUCs in 2D SWE (*p* = 0.0038) and 3D SWE (*p* = 0.0057). Moreover, the false-negative mass (category 3) was still false negative after adding E_SD_^U^ in both 2D and 3D SWE. Previous studies have shown inconsistent elastic modulus measurements. Evans *et al*.^29^ placed an ROI in the stiffest part of the SWE image, whereas Zhou *et al*.^30^ adjusted a round ROI to the lesion contours to encompass the maximum lesion area. In our study, one round ROI was adjusted to the mass contours on SWE images and another was adjusted to the mass contours on greyscale images, ensuring that mass heterogeneity could be depicted (Fig. 1). Then, E_SD_^E^ and E_SD_^U^ were measured. Previous studies have shown that tumour-associated stiffness shown by SWE was usually located in the surrounding stroma rather than in the cancerous tissue itself^18^,^29^. Due to the attenuation of the shear wave energy in the peritumoural region^36^ or the low shear wave amplitude and/or noise within the malignant lesion^37^, the shear wave may not be measurable in these malignant masses. Therefore, the heterogeneity reflected by E_SD_^U^, which does not include peritumoural stiffness observed by SWE imaging, may be inaccurate. Moreover, the surrounding desmoplastic reaction and infiltration of the cancer cells into interstitial tissues may be responsible for the stiff zone surrounding the mass on SWE^25^,^29^. The stiff rim sign proposed by Zhou *et al*.^30^ has shown the best diagnostic performance among all SWE features. As a result, E_SD_^E^ that includes the mass and immediate adjacent stiff tissue or halo can better illustrate the heterogeneity and thus has a better diagnostic value, especially for malignant masses.

**Figure 4:**
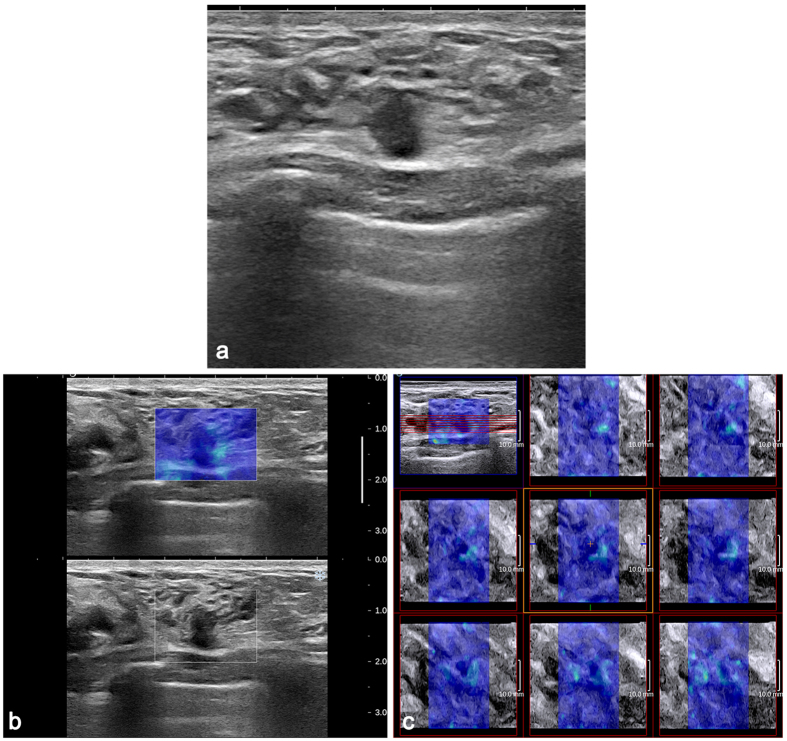
DCIS in a 36-year-old woman. (**a**) The 2D B-mode image shows a 7.4-mm, irregular hypoechoic mass with an uncircumscribed margin considered to be BI-RADS category 4a. Both 2D SWE (**b**) and 3D SWE (**c**) show a homogeneously soft (blue colour) mass and surrounding tissue (2D E_max_ 58.7 kPa, E_SD_^E^ 10.2 kPa, E_SD_^U^ 9.4 kPa; 3D E_max_ 87.1 kPa, E_SD_^E^ 11.8 kPa, E_SD_^U^ 9.5 kPa). When combining SWE with B-mode US, BI-RADS category 3 was assigned to the mass.

Our results demonstrate that adding SWE features, especially E_SD_^E^, to conventional US data is significantly effective for reducing unnecessary interventional procedures. In the benign group, interventional procedures could be avoided in 37.9% (52/137) of the masses after adding the E_SD_^E^ of 3D SWE and in 39.4% (54/137) of the masses after adding the E_SD_^E^ of 2D SWE. Recent studies have been focused on the potential of elastography to reduce the number of biopsy procedures performed on benign masses. Lee *et al*.^28^ also demonstrated that the elastographic features from 2D and 3D SWE could increase the specificity of US-based diagnosis and decrease the negative biopsy rates. Athanasiou *et al*.^18^ reported that by adding quantitative SWE measurements to BI-RADS category 4 lesions, biopsies could be avoided in 46% of the cases. Our results were consistent with the findings of those studies. However, in our data, the biopsy decisions of two masses (category 4a) were changed to follow-up decisions (category 3) after 2D and 3D SWE features were added. The two masses were histopathologically identified as DCIS and were smaller than 1 cm. Other studies have shown that DCIS lesions were softer than invasive cancers^8^, and soft cancers are frequently small (≤10 mm), screen detected and low grade^38^. On the B-mode sonograms, the two malignant masses had suspicious morphological features, were irregular and were not circumscribed (Fig. 4). Therefore, whether on 3D or 2D SWE, elastography evaluations should not override the morphological features of malignancies observed on B-mode sonograms, especially for small masses. A comprehensive analysis and further studies with larger populations are of great necessity.

There were several limitations to this study. First, in our study, a multivariate analysis for evaluating confounding factors was not performed. Elasticity values were analysed according to the benign or malignant nature of the breast masses. However, some factors, such as the influences of histological differentiation, lesion size, the existence of microcalcification, the mass depth and the breast thickness, which are widely accepted to influence diagnostic performance^39^, were not evaluated. Second, selection bias may affect the results of our study because of its retrospective nature and patients being scheduled for biopsy. Third, the inter- and intraobserver variabilities for the elasticity measurements were not evaluated. According to the study design, the images were reviewed by two radiologists together, and disagreements were resolved by consensus. Moreover, a high reproducibility for SWE has previously been reported by several authors, including Cosgrove *et al*.^19^, Athanasiou *et al*.^18^ and Youk *et al*.^27^. Therefore, we assume that the inter- and intraobserver variabilities may not greatly affect the results of our study.

In conclusion, adding quantitative elasticity to B-mode US improved performance and specificity and reduced the occurrence of false-positive biopsies. Compared with E_max_, the measurement of SD, especially E_SD_^E^, should be more stable given the lower effect of artefacts on 3D SWE. In addition, the measurement of E_SD_^E^ showed better clinical value than that of E_SD_^U^.

## Additional Information

**How to cite this article**: Tian, J. *et al*. Application of 3D and 2D quantitative shear wave elastography (SWE) to differentiate between benign and malignant breast masses. *Sci. Rep.* **7**, 41216; doi: 10.1038/srep41216 (2017).

**Publisher's note:** Springer Nature remains neutral with regard to jurisdictional claims in published maps and institutional affiliations.

## Figures and Tables

**Table 1:** Medians (IQRs) of elasticity values in kilopascals (kPa) of malignant and benign masses measured on 2D and 3D SWE acquisitions.

	Benign	Malignant	*p* value
2D SWE			
E_SD_^U^	6.40 (4.37–8.43)	15.50 (11.63–24.07)	<0.0001
E_SD_^E^	6.80 (4.93–9.30)	21.63 (16.20–27.83)	<0.0001
E_max_	42.23 (30.03–51.67)	139.90 (108.80–177.53)	<0.0001
3D SWE			
E_SD_^U^	7.00 (5.23–8.80)	16.23 (11.43–24.97)	<0.0001
E_SD_^E^	7.70 (6.00–10.30)	20.27 (16.53–27.20)	<0.0001
E_max_	78.00 (48.00–110.70)	185.40 (154.90–220.00)	<0.0001

2D = two-dimensional, 3D = three-dimensional, SWE = shear wave elastography, IQR = interquartile range.

**Table 2:** Diagnostic performances of 2D and 3D SWE for distinguishing benign from malignant breast masses.

	AUC			Sensitivity			Specificity		
	2D	3D	*p* value	2D	3D	*p* value	2D	3D	*p* value
E_SD_^U^	0.9142	0.8891	0.159	58/73 (79.45)	62/73 (84.93)	0.387	126/137 (91.97)	116/137 (84.67)	0.060
E_SD_^E^	**0.9553**^**#**^	0.9306*	**0.026**	65/73 (89.04)	61/73 (83.56)	0.336	131/137 (95.62)	127/137 (92.70)	0.303
E_max_	**0.9636**^**&**^	0.9252	**0.002**	66/73 (90.41)	65/73 (89.04)	0.785	133/137 (97.08)	116/137 (84.67)	**0.000**

The numbers in parentheses are proportions. *p = 0.0057, comparing the AUC of E_SD_^U^ and E_SD_^E^ on 3D SWE. ^#^*p* = 0.0038, comparing the AUC of E_SD_^U^ and E_SD_^E^ on 2D SWE. ^&^*p* = 0.0016, comparing the AUC of E_SD_^U^ and E_SD_^max^ on 2D SWE.

**Table 3:** Diagnostic performances of B-mode US and B-mode US combined with SWE.

Parameter	Threshold*	AUC	*p* value^#^	Sensitivity	Specificity	*p* value^*^	PPV	NPV
B-mode US category^a^	>4a	0.9064	NA	67/73 (91.78)	110/137 (80.29)	NA	67/94 (71.28)	110/116 (94.83)
+2D SWE								
E_SD_^U^	>10.73	0.9571	0.0005	63/73 (86.30)	130/137 (94.89)	0.0002	63/70 (90.00)	130/140 (92.86)
E_SD_^E^	>13.4	0.9683	0.0003	66/73 (90.41)	132/137 (96.35)	<0.0001	66/71 (92.96)	132/139 (94.96)
E_max_	>80.8	0.9767	<0.0001	65/73 (89.04)	132/137 (96.35)	<0.0001	65/70 (92.86)	132/140 (94.29)
+3D SWE								
E_SD_^U^	>10.23	0.9454	0.0007	60/73 (82.19)	129/137 (94.16)	0.0006	60/68 (88.24)	129/142 (90.85)
E_SD_^E^	>13	0.9563	0.0007	62/73 (84.93)	131/137 (95.62)	<0.0001	62/68 (91.18)	131/142 (92.25)
E_max_	>131.7	0.9562	0.0016	66/73 (90.41)	122/137 (89.05)	0.0442	66/81 (81.48)	122/129 (94.57)

^a^B-mode US category was defined according to the BI-RADS classification for US. NA = not available. The + before 2D and 3D SWE indicates the effect of adding that SWE feature to the original BI-RADS category 4a and 4b masses. ^#^*p* value of testing the null hypothesis that there is no change in AUC with the addition of the SWE feature (McNemar test). *p value of testing the null hypothesis that there is no change in specificity with the addition of the SWE feature (McNemar test). *Threshold as the optimal cut off, yielding the maximal sum of the sensitivity and specificity.

**Table 4:** Estimates of the effect of 2D and 3D SWE features on the biopsy recommendation for breast masses.

	BI-RADS 3		BI-RADS 4a		Biopsy recommendation in the benign group	*p* value	Biopsy recommendation in the malignant group	*p* value
	benign masses	malignant masses	benign masses	malignant masses				
B-mode	46	1	57	5	84/137 (61.31)	NA	72/73 (98.63)	NA
+2D SWE								
E_SD_^U^	95	3	8	3	35/137 (25.55)	<0.0001	70/73 (95.89)	0.6198
E_SD_^E^	100	2	3	4	30/137 (21.89)	<0.0001	71/73 (97.26)	1.0000
E_max_	101	3	2	3	29/137 (21.16)	<0.0001	70/73 (95.89)	0.6198
+3D SWE								
E_SD_^U^	89	3	14	3	41/137 (29.92)	<0.0001	70/73 (95.89)	0.6198
E_SD_^E^	98	2	5	4	32/137 (23.35)	<0.0001	71/73 (97.26)	1.0000
E_max_	92	2	11	4	38/137 (27.73)	<0.0001	71/73 (97.26)	1.0000

The numbers in parentheses are percentages. NA = not available. The + before 2D and 3D SWE indicates the effect of adding that the SWE feature to the original BI-RADS category 3 and 4a masses.
